# Adapting and Surviving: Intra and Extra-Cellular Remodeling in Drug-Resistant Gastric Cancer Cells

**DOI:** 10.3390/ijms20153736

**Published:** 2019-07-31

**Authors:** Sabino Russi, Henu Kumar Verma, Simona Laurino, Pellegrino Mazzone, Giovanni Storto, Anna Nardelli, Pietro Zoppoli, Giovanni Calice, Francesco La Rocca, Alessandro Sgambato, Valeria Lucci, Geppino Falco, Vitalba Ruggieri

**Affiliations:** 1 Laboratory of Preclinical and Translational Research, IRCCS-Referral Cancer Center of Basilicata (CROB), 85028 Rionero in Vulture (PZ), Italy; 2 Institute of Experimental Endocrinology and Oncology, National Research Council, 80131 Naples, Italy; 3 Section of Stem Cell and Development, Istituto di Ricerche Genetiche “Gaetano Salvatore” Biogem s.c. a.r.l., 83031 Ariano Irpino, Italy; 4 Department of Nuclear Medicine, IRCCS—Referral Cancer Center of Basilicata (CROB), 85028 Rionero in Vulture (PZ), Italy; 5 Istituto di Biostrutture e Bioimmagini, Consiglio Nazionale delle Ricerche, 80145 Napoli, Italy; 6 Laboratory of Clinical Research and Advanced Diagnostics, IRCCS-Referral Cancer Center of Basilicata (CROB), 85028 Rionero in Vulture (PZ), Italy; 7 Scientific Direction, IRCCS-Referral Cancer Center of Basilicata (CROB), 85028 Rionero in Vulture (PZ), Italy; 8 Department of Biology, University of Naples Federico II, 80126 Naples, Italy

**Keywords:** gastric cancer, drug resistance, cellular reprogramming, cell behavior

## Abstract

Despite the significant recent advances in clinical practice, gastric cancer (GC) represents a leading cause of cancer-related deaths in the world. In fact, occurrence of chemo-resistance still remains a daunting hindrance to effectiveness of the current approach to GC therapy. There is accumulating evidence that a plethora of cellular and molecular factors is implicated in drug-induced phenotypical switching of GC cells. Among them, epithelial-mesenchymal transition (EMT), autophagy, drug detoxification, DNA damage response and drug target alterations, have been reported as major determinants. Intriguingly, resistant GC phenotype may be the result of GC cell-induced tumor microenvironment (TME) remodeling, which is currently emerging as a key player in promoting drug resistance and overcoming cytotoxic effects of drugs. In this review, we discuss the possible mechanisms of drug resistance and their involvement in determining current GC therapies failure.

## 1. Introduction

In the last decade, remarkable progress has been made in understanding the complex molecular mechanisms responsible for the onset and progression of human gastric cancer (GC). It represents the second most common cause of cancer deaths worldwide [1]. The incidence of GC varies significantly among different geographical areas, with higher rates in Eastern Asia, European and South America Countries and lower ones in North America and Africa regions [2,3].

To date, the most common therapeutic options for GC remain surgery and/or chemotherapy [4], although only surgical resection is considered curative, ensuring a five-year overall survival rate of 60–80% [5], in the case of an early stage diagnosis [6].

Several chemotherapeutic strategies have been used in patients with unresectable tumors to relieve symptoms and decrease the risk of recurrence [7] and metastasis [8,9], with a 5-year overall survival of 20–35% [10,11]. Moreover, compared with surgery alone, perioperative chemotherapy is able to significantly improve prognosis of patients with resectable GC [12,13]. However, at present, in 70–90% of cases, chemotherapy is unable to inhibit tumor cell growth [14], cancer cell invasiveness and metastasis spread, leading to drug resistance [15,16,17,18].

Chemo-resistance of tumor cells occurs through two universally accepted mechanisms [19]: It may be already pre-existent at diagnosis, meaning that tumor cells are intrinsically resistant to the chemotherapeutic agent [20], or it can be induced after the exposure of cancer cells to the drug [21,22,23]. These two resistance profiles are defined as intrinsic and acquired resistance, respectively, and they are both related to tumor cells, as well as to tumor microenvironment (TME) characteristics [24,25]. Hence, elucidation of the molecular mechanisms underlying drug resistance is of paramount importance in order to improve GC patients’ survival. Nevertheless, the molecular mechanisms of drug resistance in GC, such as involvement of host factors [26], substantial changes in genetic and epigenetic factors [27,28], and mutations in drug targets, have been only partially defined [29].

Here, we reviewed the recent literature on the molecular mechanisms by which GC cells promote intra and extra-cellular remodeling for overcoming anticancer drugs effects.

## 2. Role of Microenvironment in Tumor Growth and Chemo-Resistance

The role of TME in tumor progression and drug resistance is an attractive area of investigation [30,31,32], mainly focusing on the molecular mechanisms underlying the TME responsiveness to anticancer drugs and the crosstalk between cancer cells and their environment [33]. The interaction between tumor cells and stromal components involves growth factors, chemokines, cytokines and non-cellular components such as the extracellular matrix (ECM), all contributing to tumor development and drug resistance in different types of cancers, including GC [34,35,36,37]. Furthermore, there is emerging evidence suggesting that the accumulation of various stromal cells (SC) such as fibroblasts, endothelial cells, adipose tissue-derived stromal cells (ATDSC), several immune cells or inflammatory cells and bone marrow-derived stem cells (BMDSC) could be involved in drug resistance through cell-to-cell communication, tumor-to-stromal cell communication [38], and tumor-to-ECM interaction [39].

### 2.1. Angiogenesis and Hypoxia

Angiogenesis is a prerequisite for a rapid expansion of the tumor cell population, favoring an increase in tumor size and intravasation [40]. It has also been hypothesized that tumor angiogenesis is primarily involved in tumor cell apoptosis inhibition and chemo-resistance. Furthermore, tumor angiogenesis is correlated with the prognosis of several tumors, including GC [41]. A primary driving force of angiogenesis is the reduction of oxygen levels, so-called hypoxia, caused by the abnormal proliferation of cancer cells and the anomalous organization of the neo-formed vasculature network [42,43]. Hypoxia leads to the expression of several genetic factors involved in tumor progression and metastasis in GC [44,45]. The most important one is hypoxia-inducible factor-1α (HIF-1α), which regulates cellular response to hypoxia [46]. HIF-1α expression has been found to be significantly associated with drug resistance in GC [47,48]. Even if HIF-1α-dependent molecular mechanisms in chemo-sensitivity have been only partially elucidated, suppression of p53 and activation of nuclear factor κB (NF-κB) seem to play a key role in hypoxia-related 5-fluorouracil (5-FU) and cisplatin (CDDP) sensitivity in human GC cells [49]. It has been reported that HIF-1α induces drug resistance to adjuvant chemotherapy with 5-FU in advanced GC patients [50]. Moreover, the overexpression of HIF-1α leads to hypoxia-induced drug resistance by increasing the expression of Bcl-2, P-glycoprotein (P-gp) and MRP1, and reducing the expression of Bax [51].

### 2.2. Angiogenesis and Growth Factors

As suggested by many reports, angiogenesis in GC is controlled by various factors released by tumor and stromal cells. Furthermore, angiogenesis is responsible for tumor chemo-resistance [52]. Vascular endothelial growth factor family members (VEGFs) and their receptors represent important tumor angiogenesis inducers in GC [53]. The VEGFs are both mitogen and survival factors. It has been reported that patients with VEGF overexpressing tumors, compared to patients with VEGF negative tumors, develop resistance to chemotherapy and show a significantly poorer prognosis and shorter survival [54,55]. A previous study also reported that VEGF family expression is significantly associated with distant metastasis [56]. Consistently, upregulated VEGF-C expression is positively correlated with chemo-resistance in human GC cell lines whereas loss of VEGF-C inhibits metastasis by inducing apoptosis in vitro and in vivo [57].

Since the characterization of the VEGF family, several other factors have been described as regulators of angiogenesis in cancer. Among these, cancer-associated fibroblasts (CAFs) represent an abundant component in TME. During tumor progression, CAFs transdifferentiate from resident fibroblasts, endothelial cells, preadipocytes and bone marrow-derived mesenchymal stem cells (MSCs) and are able to deeply impact cancer behavior and anti-cancer treatment efficacy [58]. Innate and adaptive properties of CAFs indeed contribute to tumor progression and metastasis, as well as to chemo-resistance occurrence in different human cancers [58,59]. Several therapeutics approaches targeting CAF have been investigated to date, resulting in decreased tumor growth and also in cancer cells enhanced immunotherapy sensitivity [60]. Until now, the phenotype characterization and mechanism of interaction between GC cells and CAFs has not yet been fully elucidated. However, it has recently been reported that CAFs produce soluble tumor-promoting factors such as SDF-1/CXCL12 and promote GC cell invasiveness by inducing integrin beta1 clustering [60]. Increase of VEGF-A expressing CAFs, derived from bone marrow, was reported in mouse GC after co-activation of prostaglandin E_2_ (PGE_2_) and Wnt signaling pathway [61]. GC cells and CAFs are also able to secrete IL-1, prostaglandin and sphingosin 1 phosphate (S1P), promoting chemo-resistance via IL-11/IL-11R/JAK/STAT3, an anti-apoptosis signaling pathway [62]. A recent study demonstrated that activated gastric CAFs correlate with poor prognosis and contribute to the increased resistance to 5-FU via paracrine action [63].

Several interleukins (ILs) have also been implicated in key angiogenic events modulation in GC. Overexpression of IL-8 in MKN45 gastric cancer cell line is responsible for increased cell–cell adhesion, cellular migration, invasion and resistance to oxaliplatin [64]. IL-33, a member of the IL-1 family, showed a close relationship with invasiveness, while IL-33 overexpression increased the activation of the JNK signaling pathway, protecting GC cells from platinum-induced apoptosis [65]. IL-6, one of the major cytokines, can induce the activation of STAT3 in GC tissue [66], and it has been found to be associated with trastuzumab resistance [67]. Tumor necrosis factor-related apoptosis-inducing ligand (TRAIL) is a TNF family member that promotes NF-κB-signaling pathway mediated 5-FU resistance in GC cells [68,69]. A proliferation-inducing ligand (APRIL) is a TNF family member and a cytokine involved in CDDP resistance in GC cells [70]. To date, several targets playing a key role in the angiogenesis-mediated chemo-resistance have been discovered. Based on this evidence, several antiangiogenic factors are currently used in the treatment of GC to overcome chemo-resistance mechanisms [71,72].

## 3. Role of Microenvironment in Tumor Growth and Chemo-resistance

Epithelial-mesenchymal transition (EMT) is recognized as a crucial process in embryonic development of multicellular organisms and wound healing. The EMT was identified as a pivotal driver in tumors initiation and progression. This often-reversible trans-differentiation process, indeed, endows originally epithelial cells with migratory mesenchymal features and also with cancer stem cell (CSC) properties, such as tumorigenicity, self-renewal, apoptosis escaping, through a multistep and finely orchestrated reprogramming [73].

Far from being a univocal process, EMT consists, instead, of a wide spectrum of transitional states in which the interplay among several molecular mechanisms, from signal transduction pathways to epigenetic changes, microRNA and growth and transcription factors, exerts a key regulatory function [74,75,76,77].

The cross-talk between extra and intra-cellular signals involved in EMT process converges at profound cellular modifications, such as down-regulation of E-cadherin, a typical epithelial marker, and up-regulation of N-cadherin and other mesenchymal proteins [76].

A large body of evidence highlights a close relationship between EMT status and cancer cell metastatic properties, chemo-resistance and worse clinical outcomes across a wide range of tumors, including GC [76,78,79,80,81,82,83,84].

One of the most recent findings demonstrated that the overexpression of miR-30 induces the occurrence of an epithelial-like phenotype associated with a higher CDDP sensitivity in mesenchymal resistant GC cells and that miR-30 is significantly down-regulated in chemo-resistant GC patients [85]. Moreover, the oncogenic protein TAZ (transcriptional co-activator with PDZ-binding motif) is highly expressed in CDDP-resistant GC cells and in GC patients. It is closely correlated with lymphatic metastasis and tumor TNM stages, and its depletion partially reverses EMT to mesenchymal-epithelial transition (MET) and sensitizes resistant cells to CDDP [86]. Furthermore, acquired resistance to lapatinib of HER2-positive GC cells has been revealed to be associated with Testican-1-mediated EMT [87], and CDDP resistance positively correlates with EMT induced by HER2 up-regulation [88]. Another recent study confirms the EMT role in CDDP chemo-resistance, demonstrating that knockdown of the translation initiation factor eIF5A2 promotes GC cells’ sensitivity to the drug by reversing the EMT process and inhibiting CDDP action on EMT-related markers [89]. The EMT process has also been associated with acquired resistance to selective FGFR inhibitors in GC cells [90] and transient doxorubicin (DOX) treatment induced a β-catenin-dependent EMT, promoting GC cells migration ability as well [91].

Many reports support the notion that EMT is strictly related to the occurrence of CSCs-like properties in GC and many other tumor types [92]. The combination of stemness and EMT is an independent prognostic factor for GC patients’ outcomes [93]. Recently, it has been reported that ectopic expression of embryonic stem cells transcription factor, NANOGP8, in GC cells, promotes sphere-forming and chemo-resistance by up-regulating EMT inducers and CSCs markers [94]. Moreover, expression of LGR5 and EMT-related genes in GC sphere cells is significantly associated with drug resistance [95]. Interestingly, targeting of gastric CSCs by the proton pump inhibitor, pantoprazole, inhibits 5-FU chemo-resistance via the EMT/β-catenin pathways [96]. With respect to the molecular mechanisms connecting EMT to the occurrence of chemo-refractory cells with CSC phenotypes, compelling evidence exists supporting a major role for TGF-β signaling and Wnt pathways [97]. It is also worth mentioning that EMT-related signaling functionally interacts with the autophagy pathway, which, as described below in more detail, is deeply intertwined with the fate of cancer cells. However, the relationship between EMT and autophagy seems to play a crucial role in cancer cells [98]. Intriguingly, autophagy exerts a dual effect on EMT, as well, by triggering or suppressing it, depending on the contextual conditions. The EMT process, in turn, has a dramatic impact on autophagy modulation, by regulating signal pathways, such as integrin, NF-κB, Wnt and TGF-β [99]. Considering the complexity of this interplay, unveiling mechanisms of their mutual regulation is challenging, but has potential clinical benefits in cancer therapy.

## 4. Role of Autophagy in GC Chemo-Resistance

Autophagy is a catabolic process that, in response to stress and starvation conditions, leads to engulfment, digestion and recycling of intra-cellular components in order to sustain cellular survival. Autophagy plays a key homeostatic and protective function both in physiological and pathological contexts [100]. Molecular mechanisms governing autophagy process, which can be divided into initiation, nucleation and elongation of autophagosome, fusion of autophagosome with the lysosome, and degradation of sequestered material, have been partially elucidated, and have also been widely exploited as novel biological targets in cancer therapies [101,102]. Autophagy is constitutively activated in cancer cells through the deregulation of PI3K/Akt/mTOR molecular axis and AMP-activated protein kinase (AMPK) signal transduction, which contributes to the metabolic reprogramming of cancer cells [103,104].

Most evidence supports a context-dependent double-edged sword role of autophagy in cancer. Autophagy on one hand, due to its damage-mitigation effect, limits tumorigenesis, while actually promoting cancer cells fitness due to its pro-survival role in stress conditions, on the other [105]. These apparently contradictory phenomena have been detected in several types of cancer, including GC, in which autophagy-related proteins, such as Beclin 1 (BECN1), microtubule-associated protein 1 light chain 3 (MAP1-LC3), and p62/sequestosome 1 (SQSTM1) have an important prognostic value. Long-term *Helicobacter pylori* infection has been reported to promote gastric tumorigenesis by dramatically impairing autophagy that, in turn, is able to modulate pathological processes, such as GC metastasis, by affecting TME [106,107]. In consideration of its multifaceted roles in sustaining cell survival, it is not surprising that autophagy acts as a protective mechanism for tumor cells in chemotherapy, promoting drug resistance as well [108].

One of the most recent pieces of evidence regarding this is the significant association found between the autophagy-related gene-5 (ATG-5) over-expression and poor overall survival in GC patients, and its involvement in CDDP chemo-resistance in vitro [109]. Furthermore, autophagy has also been identified as one of the molecular mechanisms by which metadherin induces 5-FU resistance in the GC MKN45 cell line [110].

Moreover, An et al. demonstrated that an ATG12-dependent autophagy regulatory loop, inhibited by miR-23b-3p, has a major role in favoring GC cells drug resistance [111]. Moreover, GC cells CDDP resistance, associated with aquaporin 3 (AQP3) over-expression, is mediated by autophagy activation and reversed by the autophagy inhibitor chloroquine [112]. Autophagic flux may also be implicated in HER2-positive human GC NCI-N87 cells to trastuzumab [113].

## 5. Multidrug Resistance (MDR) Mechanisms in GC

Multidrug resistance (MDR) consists of different mechanisms that make cancer cells resistant to several structurally and mechanistically unrelated drugs at the same time. MDR occurs as a selection process of a cancer cell population during the administration of an anticancer agent. Widespread studies have been carried out to reveal the molecular mechanisms of drug resistance in cancer cells, which fall in two main categories: (a) drug-targeted mechanisms (changes in uptake, efflux, and metabolism of anticancer agents), and (b) drug cytotoxic effect compensation mechanisms (drug target mutation or expression modulation, cell cycle arrest, increased DNA repair, reduced apoptosis, etc.).

Regarding GC, several studies have investigated the mechanisms responsible for MDR and identified several genes in drug-resistant GC cell lines. Among these, many are different from those reported for hematopoietic or other solid tumors. For example, Zhao et al. reported a set of genes differentially expressed in two drug-resistant human gastric adenocarcinoma cell lines, SGC7901/VCR (resistant to vincristine) and SGC7901/ADR (resistant to adriamycin), as compared with their parental cell line SGC7901 [114]. Below, we reported the state of art in knowledge of MDR mechanisms in GC.

### 5.1. Role of ATP-Binding Cassette (ABC) Transporters

Increased drug efflux is a MDR mechanism that involves ATP-binding cassette (ABC) transporters that physiologically play a major role in the transport of nutrients and other molecules across the membrane. It has been demonstrated that ABC transporters are often overexpressed in GC tumors and associated with chemo-resistance. P-glycoprotein (P-gp or MDR-1 or ABCB1) is one of the most investigated ABC transporters, and was found to be overexpressed in GC and associated with a shorter survival in GC patients [115,116]. With respect to the correlation between P-gp and GC chemo-resistance, controversial results have been reported. These high expression levels were not, indeed, predictive of a poor prognosis in GC patients treated with 5-FU and DOX-based adjuvant chemotherapy [117]. P-gp was also determined to be dispensable for MDR occurrence in GC cell lines [118] and gastric tissue samples [119]. On the contrary, Chung et al. reported that P-gp expression rate increased from 27.8% to 37.5% pre to post administration of DOX, and correlated with a higher rate of systemic recurrence of GC [120]. Interestingly, targeting of Wnt/β-catenin pathway, which directly controls P-gp expression, induced P-gp levels reduction and MDR reversion in GC cells [121]. Similar scenarios have been found in GC samples expressing the transcriptional factor NRF2, which induces P-gp expression. NRF2 expression was also found to strongly correlate with tumor size, histological grade, lymph node, and distant metastasis [122].

Other ABC transporters, such as ABCC1 and ABCC2, are associated with MDR in GC. Indeed, Xu et al. showed that ABCC1 and P-gp positive expression rates were significantly higher in primary gastric cancer GC cells resistant to DOX, etoposide (VP-16), and hydroxycamptothecin (HCPT) [123]. However, it was found that both GC and the adjacent normal mucosa express high levels of ABCCl protein [124]. Furthermore, in advanced GC patients treated with 5-FU and DOX-based adjuvant chemotherapy, ABCC1 expression did not predict poor prognosis [117]. On the other hand, Li et al. reported that the ABCC2 polymorphism rs717620 genotypes were associated with different response to neoadjuvant chemotherapy in a cohort of advanced GC patients treated with oxaliplatin and fluoropyrimidines [125]. Patients with CC genotype had poorer outcomes and responded 3.8 times less to chemotherapy than those with TT and TC genotypes.

Solute carriers (SLCs) are membrane transport proteins whose expression is known to be associated with sensitivity to chemotherapy as for SLC29A1 and SLC22A2, which play a critical role in the uptake of gemcitabine and 5-FU, respectively [126,127]. Shimakata el al. observed that Alfa-fetoprotein (AFP)-producing GC expresses both SLC29A1 and SLC22A2 at high levels, thus suggesting that patients with this aggressive subtype of GC could benefit from gemcitabine/5-FU combination therapy [128].

### 5.2. Increased Drug Detoxification

The Glutathione S-transferases (GSTs) is a family of enzymes that plays a pivotal role in cellular detoxification against a variety of xenobiotics and noxious compounds by catalyzing their conjugation with reduced glutathione (GSH) [129]. The expression levels of GST were significantly higher in GC compared to normal gastric mucosa [130]. In addition, GST overexpression has been suggested to be involved in CDDP resistance in MKN45 GC cell line [131]. Among patients treated with fluorinated pyrimidines and mitomycin C (MMC)-based chemotherapy, the GST-pi-negative group showed a higher rate of 5-years disease-free survival (DFS) than the GST-pi-positive group [132]. Indeed, primary single-cell suspensions resistant to 5-FU, CDDP and MMC, isolated from primary gastric cancers, express high levels of GST-pi [133]. In contrast, it was found that the GSH, GST activity and GST-pi levels before the start of therapy were not predictive of response. However, GSH and GST parameters were found increased in GC patients experiencing a response to chemotherapy as compared with progressive patients [134]. Similarly, slightly increased GST-pi expression was observed in gastric tumor samples compared with adjacent normal gastric mucosa and this GST-pi expression did not correlate with CDDP resistance [135]. The inconsistent results regarding the role of GST-pi in GC chemo-sensitivity imply that many additional factors are involved in MDR and the effective role of GST-pi in GC drug resistance needs to be clarified.

Synthesis of reduced GSH is also promoted by the interaction of CD44 splice variants (CD44v) with the xCT subunit of the cystine-glutamate exchange transporter (xc-) and is associated with resistance to oxidative stress [136,137]. This mechanism could explain the association between CD44v expression and high recurrence risk in GC patients [138,139]. Two clinical trials by Shitara et al. showed that the inhibition of xc- system by sulfasalazine (SSZ) reduces the levels of CD44v-positive cells and GSH in some GC patients, even in those refractory to CDDP [140,141].

### 5.3. Counteracting Drug-Induced DNA Damage Response

The main desirable effect of chemotherapeutic drugs is to induce massive cell death in tumor cells by apoptosis. A body of evidence indicates that chemo-resistance is possibly due to a defective apoptotic pathway. p53 is a tumor suppressor protein whose inactivation could be associated with resistance to chemotherapy in GC cells. Indeed, wild-type p53 expression in GC cell lines is significantly associated with response to CDDP and 5-FU as compared with mutant p53 expression [142]. Another study reported a significant up-regulation of p27 and a down-regulation of p53 and p21 in GC cell lines chronically exposed to CDDP. These results indicate a possible role for p27-dependent cell cycle arrest in CDDP-induced apoptosis escaping in GC cells [143]. Treatment with the recombinant adenoviral vector encoding the wild-type human tumor-suppressor protein p53 gene (rAd-p53) inhibited the growth of three GC cell lines and synergistically enhanced their sensitivity to oxaliplatin [144]. However, the response rate of patients with p53-negative locally advanced GC to epi-doxorubicin or 5-FU-based chemotherapy was found to be significantly higher when compared to patients with overexpression of p53 [145]. Despite these contrasting results, a recent meta-analysis demonstrated that p53 expression is positively linked to a better chemotherapy response in GC patients [146]. In a GC cell line, it was also demonstrated that the administration of parthenolide reverses drug-resistance to CDDP by inducing p53 expression [147]. Moreover, it was found that overexpression of homeobox A13 (HOXA13) gene makes GC cells more resistant to 5-FU by a p53-dependent pathway [148]. Similarly, overexpression of inhibition of apoptosis-stimulating protein of p53 (iASPP) was reported in GC patients, as well as in CDDP-resistant GC cell lines [149]. In response to DNA damage, p53 can bind epsin 3 (EPN3) promoter and induce its expression. EPN3 was found down-regulated in GC samples, and its knock-down resulted in resistance to DNA damage-induced apoptosis [150]. Notably, G2 and S-Phase Expressed 1 (GTSE1) gene, a p53 activity repressor, was found to be dose-dependently induced by CDDP treatment in GC cells and its knock-down enhanced p53-mediated apoptosis in response to CDDP [151]. NF-κB also plays a role in compensating p53 activity. Indeed, it was shown that 5-FU treatment determines the NF-κB-dependent up-regulation of different p53 targets and that cell lines bearing Pro/Pro homozygosity at codon 72 of p53 exon 4 are more prone to 5-FU resistance [152]. Another strategy of GC cells for avoiding apoptosis pathway is that of regulating intracellular Ca^2+^ levels by the modulation of the expression and/or the activity of a multitude of Ca^2+^ channels and transporter proteins. We recently investigated this issue, and we found that the transient receptor potential cation channel subfamily V member 2 (TRPV2) is up-regulated in GC samples when compared with normal ones. TRPV2 expression levels are associated with worse prognosis in both overall and adjuvant-treated patients [153].

The acquisition of MDR can derive from the induction of DNA repair machinery, as hypothesized for the suppression of bridging integrator 1 (BIN1) protein by c-Myc overexpression, which gains DNA repair activity and confers CDDP resistance to GC cells [154]. The chemo-resistance prediction capability of the up-regulation of two nucleotide excision repair (NER) machinery genes was documented in a phase II study. Chemo-resistance prediction accuracy was 77.5% for damage DNA binding protein complex subunit 2 (DDB2), 75.0% for excision repair cross-complementing 1 (ERCC1), and 82.5% when combined in GC patients treated with docetaxel, CDDP, and S-1 combination chemotherapy [155]. Furthermore, Ning et al. found that DNA double-strand breaks, induced by anticancer drugs, increase telomeric repeat binding protein 2 (TRF2) expression before ATM- and p53-dependent DNA damage response activation [156]. Moreover, increased telomerase reverse transcriptase (hTERT) was found in GC samples when compared with their matched normal mucosa, and its upregulation was associated with resistance to 5-FU and DOX in GC cells [157].

### 5.4. Compensation of Drug Activity by Modulation of Targets Expression

An alternative mechanism by which GC cells can escape from drug-induced cell death is represented by the up- or down-regulation of drug targets. In this scenario, the alteration in methylation pattern is relevant for response to treatment and patient survival. DNA methyltransferases (DNMTs) modulate the expression of genes involved in cell cycle regulation, genomic instability, EMT, apoptosis and tumor suppression [158]. Moreover, Topoisomerase II (Topo II), a nuclear enzyme involved in DNA replication, is an example of a drug target whose expression is often found down-regulated in GC. Indeed, it was demonstrated that in GC cells resistant to HCPT, DOX and MMC, Topo II down-regulation impaired cross-linked DNA formation [133]. The opposite mechanism involves the overexpression of thymidylate synthase (TS) enzyme, on which 5-FU acts, avoiding its binding to the natural substrate. Indeed, higher TS expression may predict drug resistance to 5-FU treated GC patients [159].

## 6. Conclusions

**Figure 1:**
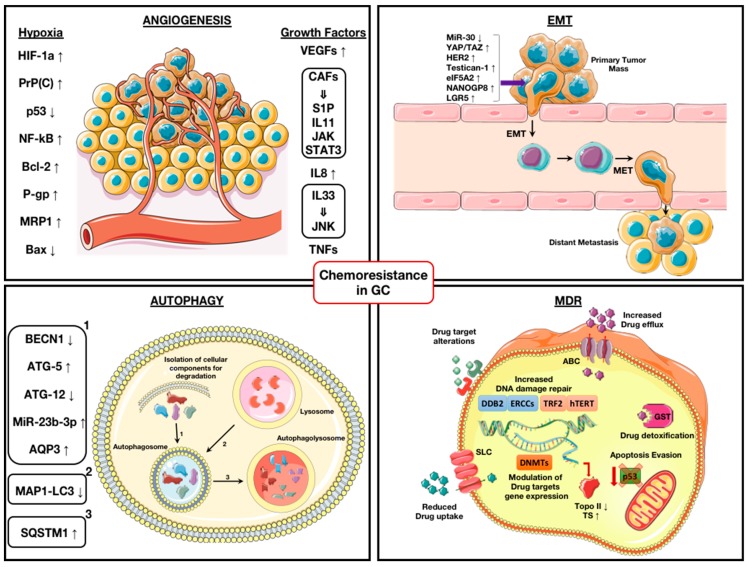
Schematic representation of the most common mechanisms involved in chemo-resistance of gastric cancer (GC) cells. **Angiogenesis:** hypoxia regulated genes and growth factors released by GC and cancer-associated fibroblasts (CAFs) implicated in chemo-resistance. **Epithelial-Mesenchymal Transition (EMT):** factors involved in epithelial-mesenchymal switch that makes cancer cells drug-resistant and able to metastasize. **Autophagy:** proteins involved in (1) isolation of cellular components for degradation in autophagosome, (2) fusion between autophagosome and lysosome, and (3) catabolic processes in autophagolysosome and found associated with chemo-resistance. **Multidrug Resistance (MDR):** proteins involved in reduced drug uptake or increased drug efflux; increased drug detoxification; mutation or down-regulation of drug targets; up-regulation of drug targets; increased drug-induced DNA damage repair or telomere maintenance pathways; epigenetic regulation of gene expression. Abbreviations: solute carrier (SLC) and ATP-binding cassette (ABC) transporters; glutathione S-transferase (GST); Topo II (Topoisomerase II); thymidylate synthase (TS); DNA methyltransferases (DNMTs). (Images built with illustrations from https://smart.servier.com under CC 3.0 license).

As for other types of cancer, drug resistance in GC is a major challenge into the clinical approach to patients. As summarized in Figure 1, several mechanisms have been implicated in the cellular and molecular modifications that make GC cells resistant to current chemotherapeutics strategies. In addition, GC cells are also able to, directly or indirectly, induce a deep and fine TME reorganization to their advantage. Deciphering and targeting these multifaceted GC cells’ remodeling processes could be useful for adapting the current chemotherapeutic drugs to the GC cells’ responsiveness, as well as in the setting up of novel tailored anticancer agents.

## Abbreviations

GC	Gastric cancer
EMT	Epithelial-mesenchymal transition
TME	Tumor microenvironment
ECM	Extracellular matrix
CDDP	Cisplatin
5-FU	F-fluorouracil
P-gp	P-glycoprotein
VEGF	Vascular endothelial growth factor
CAFs	Cancer-associated fibroblasts
ILs	Interleukins
CSCs	Cancer stem cells
MET	Mesenchymal-epithelial transition
DOX	Doxorubicin
MDR	Multidrug resistance
ABC	ATP-binding cassette transporters
HCPT	Hydroxycamptothecin
SLCs	Solute carrier transporters
GSH	Glutathione
Topo II	Topoisomerase II
